# Endothelial cell malignancies: new insights from the laboratory and clinic

**DOI:** 10.1038/s41698-017-0013-2

**Published:** 2017-04-20

**Authors:** Michael J. Wagner, Vinod Ravi, David G. Menter, Anil K. Sood

**Affiliations:** 1 Division of Cancer Medicine, The University of Texas MD Anderson Cancer Center, 1515 Holcombe Boulevard, Houston, TX 77030 USA; 2 Department of Sarcoma Medical Oncology, The University of Texas MD Anderson Cancer Center, 1515 Holcombe Boulevard, Houston, TX 77030 USA; 3 Department of Gastrointestinal Medical Oncology, The University of Texas MD Anderson Cancer Center, 1515 Holcombe Boulevard, Houston, TX 77030 USA; 4 Department of Gynecologic Oncology and Reproductive Medicine, The University of Texas MD Anderson Cancer Center, 1515 Holcombe Boulevard, Houston, TX 77030 USA; 5 Center for RNA Interference and Non-Coding RNA, The University of Texas MD Anderson Cancer Center, 1515 Holcombe Boulevard, Houston, TX 77030 USA; 6 Department of Cancer Biology, The University of Texas MD Anderson Cancer Center, 1515 Holcombe Boulevard, Houston, TX 77030 USA

## Abstract

Endothelial cell malignancies are rare in the Western world and range from intermediate grade hemangioendothelioma to Kaposi sarcoma to aggressive high-grade angiosarcoma that metastasize early and have a high rate of mortality. These malignancies are associated with dysregulation of normal endothelial cell signaling pathways, including the vascular endothelial growth factor, angiopoietin, and Notch pathways. Discoveries over the past two decades related to mechanisms of angiogenesis have led to the development of many drugs that intuitively would be promising therapeutic candidates for these endothelial-derived tumors. However, clinical efficacy of such drugs has been limited. New insights into the mechanisms that lead to dysregulated angiogenesis such as mutation or amplification in known angiogenesis related genes, viral infection, and chromosomal translocations have improved our understanding of the pathogenesis of endothelial malignancies and how they evade anti-angiogenesis drugs. In this review, we describe the major molecular alterations in endothelial cell malignancies and consider emerging opportunities for improving therapeutic efficacy against these rare but deadly tumors.

## KeyPoints

- Intermediate- and high-grade endothelial cell malignancies are rare, but can be associated with substantial morbidity and mortality.
- HIV and KSHV viral proteins directly interact with Notch pathway and VEGF pathway proteins, contributing to the tumorigenesis of Kaposi sarcoma.
- Angiosarcomas contain multiple abnormalities that may lead to primary resistance to VEGF/VEGFR inhibitors. These include *VEGFR2* (*KDR*) and *PLCG1* mutations, loss-of-function *PTPRB* mutations, and amplification of *c-MYC* and *VEGFR3 (FLT4)*.
- Treating chemotherapy-resistant endothelial malignancies with VEGF-targeted drugs has had limited success. Therefore, more effective therapies are needed.
- Understanding the deregulated signaling pathways in endothelial cell neoplasms may reveal insights into driver and drug-resistance mechanisms.

## Introduction

**Fig. 1:**
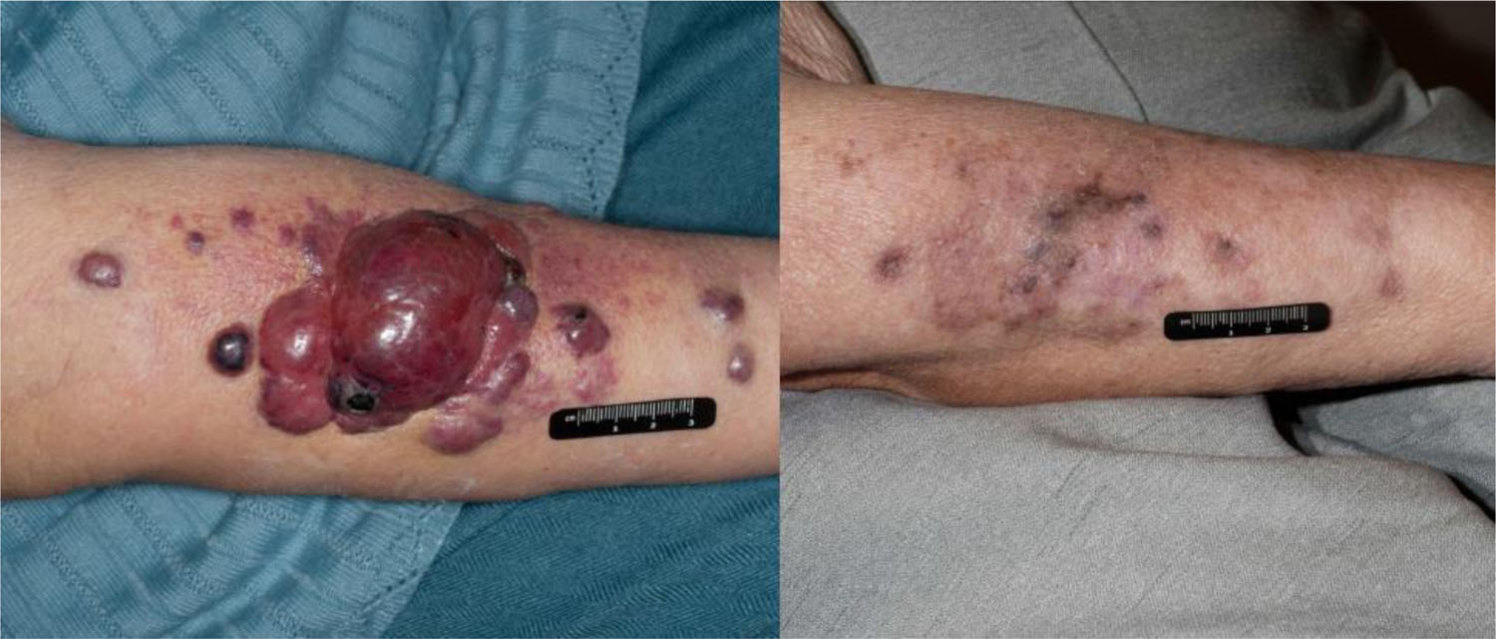
Clinical appearance of angiosarcoma and response to paclitaxel. Pretreatment appearance of cutaneous angiosarcoma in a patient with lymphedema of the upper extremity following treatment for breast cancer (*left*), and appearance after response to paclitaxel (*right*)

Endothelial cells are known to line established blood vessels and initiate the establishment of new blood and lymph channels in vascular development. Malignancies arising from endothelial cells are rare in developed countries; however, the tumors that do arise tend to be highly aggressive and difficult to treat. While high-grade endothelial malignancies respond well to traditional chemotherapy agents such as taxanes (Fig. 1), their durability is poor and the tumors acquire drug resistance rapidly.^1^ Targeted therapies such as anti-angiogenic agents that would be intuitive for these malignancies have had limited success in the clinic. Therefore, a renewed effort aimed at identifying the mechanisms by which endothelial cell malignancies evade currently available angiogenesis inhibitors is required to improve outcomes of patients with these rare diagnoses. New insights from the laboratory include identification of potential drivers of endothelial malignancies that can function as new targets for future clinical development.

The clinical and pathological features of endothelial cell malignancies have been reviewed previously.^2–5^ These tumors generally occur in adults and are classified as having intermediate or high malignant potential. Intermediate grade vascular neoplasms include epithelioid, spindle cell, psuedomyogenic, and malignant endovascular papillary hemangioendotheliomas. Each of these entities is classically differentiated by histological appearance, but recent evidence suggests that at least some of the intermediate grade hemangioendotheliomas are driven by chromosomal translocations (Table 1). Compared to other intermediate types, epithelioid hemangioendotheliomas (EHEs) of visceral origin tend to be multifocal with propensity for metastasis despite their slow proliferative rate. Histopathologically, these tumors are characterized by an epithelioid appearance with disorganized vascular channels and are sometimes mistaken for carcinomas (e.g., lung adenocarcinoma).^6^ The diagnosis is often made by the presence of one of several characteristic chromosomal translocations.

**Table 1:** Chromosomal translocations in hemangioendotheliomas and angiosarcoma

Rearrangement	Involved gene(s)	Resultant phenotype	Reference no.
t(1;3)(p36.23;q25.1)	*WWTR1, CAMTA1*	EHE	Tanas *et al*.,^158^ Anderson *et al*.,^159^ Errani *et al*.,^160^Patel *et al*.^161^
t(11;X)	*YAP1, TFE3*	EHE	Antonescu *et al*.^162^
t(10;14)	*PIGF*	EHE	He *et al*.^165^
t(7;19)(q22;q13) fusion	*SERPINE1*, *FOSB*	PHE	Walther *et al*.^166^
11p11.2-11q12.1	*NUP160, SLC43A3*	Angiosarcoma	Shimozono *et al*.^169^

*WWTR1* WW domain containing transcription regulator 1, *CAMTA1* calmodulin binding transcription activator 1, *EHE* epithelioid hemangioendothelioma, *YAP1* the yes-associated protein 1, *TFE3* transcription factor binding to IGHM enhancer 3, *PlGF* placental growth factor, *SERPINE1* serpin peptidase inhibitor, clade E (nexin, plasminogen activator inhibitor type 1) member 1, *FOSB* FBJ murine osteosarcoma viral oncogene homolog B, *PHE* pseudomyogenic hemangioendothelioma, *NUP160* nucleoporin 160 kDa, *SLC43A3* solute carrier family 43, member 3

Endothelial cell malignancies of high malignant potential include angiosarcomas and, in immunocompromised hosts, Kaposi sarcoma (KS). Angiosarcomas belong to the high-grade end of the spectrum, with an aggressive clinical course characterized by a high propensity for both local recurrence and distant metastasis. Clinically, there are two distinct subtypes. Primary angiosarcomas can occur anywhere in the body; the more common sites include the scalp, breast, liver, spleen, bone, and heart.^2^ Secondary angiosarcomas arise from chronic lymphedema in the extremities or from radiation exposure to the chest wall following breast cancer treatment and are often molecularly associated with amplification of *c-MYC*.^7^ KS has a viral etiology and is caused by KS-associated herpes virus (KSHV; also known as HHV8). Originally described in elderly men of Mediterranean descent, it is also associated with the human immunodeficiency virus (HIV) and immune suppression^8^ and is now one of the most common malignancies in Sub-Saharan Africa.^9^

**Fig. 2:**
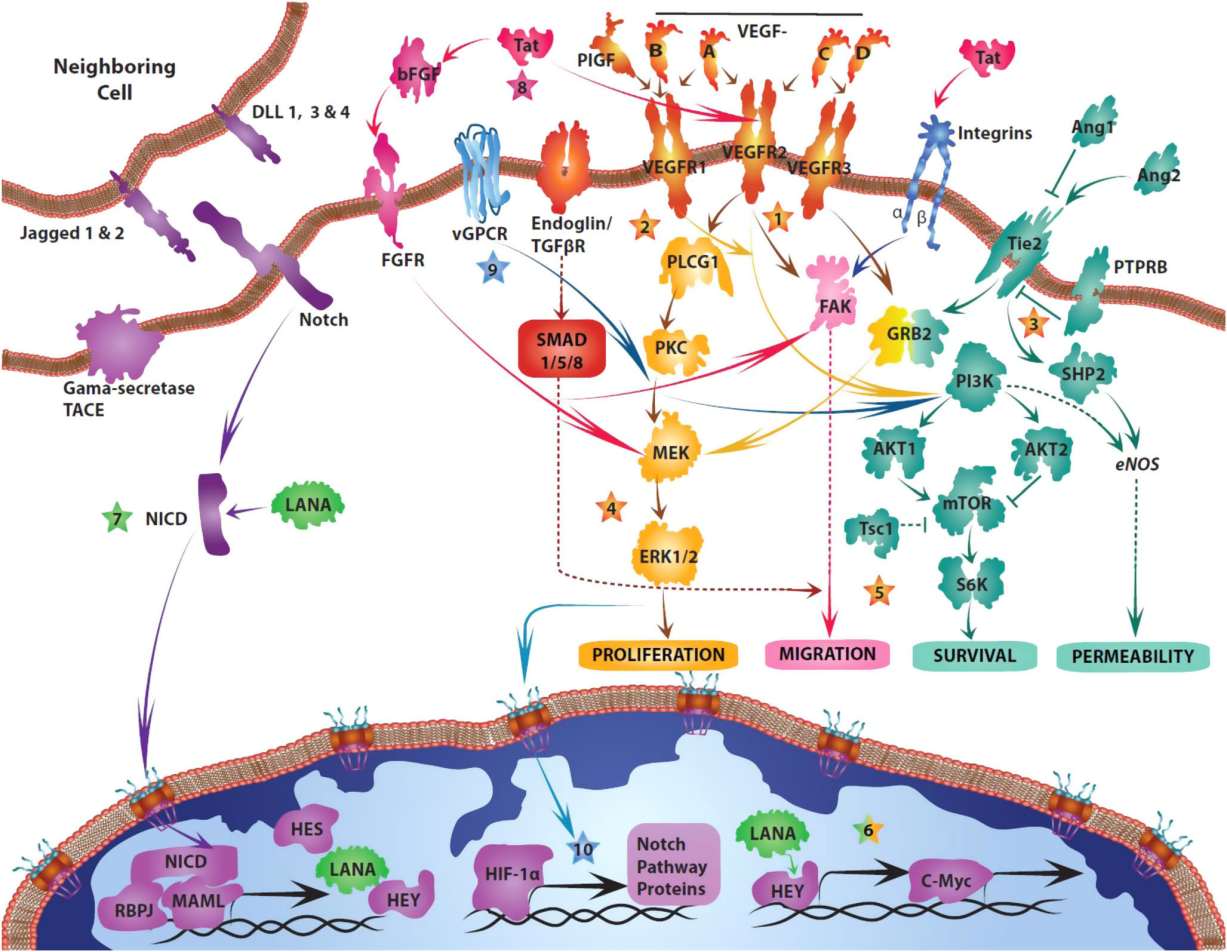
Key pathways in endothelial cell malignancies. Alterations in angiosarcoma (*orange stars*) include 1. Activating mutations in *VEGFR2* (*KDR*), and amplification of *VEGFR3* (*FLT4*), 2. A recurrent activating R707Q mutation in *PLCG1*, 3. Loss of function mutations in PTPRB, removing the inhibitory signal on Tie2, 4. Mutations in K-, *H*-, and *N-RAS*, *BRAF*, and *MAPK1*, and amplification of *B-* and *C- RAF*, and *MAPK1*. 5. Mice with loss of Tsc1 have constitutive activation of mTOR signaling and develop angiosarcoma. 6. C-MYC amplification is associated with radiation or lymphedema-induced angiosarcoma. The KSHV-derived LANA protein stabilizes HEY (6) leading to c-MYC transcription in KS cells, and stabilizes the Notch intracellular domain (NICD) (7), leading to increased Notch-mediated signaling. The HIV-1 protein Tat binds to alpha-5-beta-1 and alpha-v-beta-3 integrin receptors and stimulates migration and invasion. Tat also stimulates the release of preformed, extracellularly bound bFGF into a soluble form that can induce vascular cell growth and prevent apoptosis, and Tat directly interacts with VEGFR2 leading to ligand independent activation of its downstream effectors (8). Expression of the lytic phase KSHV viral G-protein coupled receptor (vGPCR) leads to activation of the MAPK and PI3K/mTOR pathways (9) which ultimately causes HIF-1a-mediated transcription of Notch-related proteins (10). *Ang2* angiopoietin2, *AKT* protein kinase B, *b-FGF* basic fibroblast growth factor, *BMP* bone morphogenetic protein, *DLL* delta-like, *ERK1/2* mitogen-activated protein kinase 1/2, *FGFR, FAK* focal adhesion kinase, *FGFR* fibroblast growth factor receptor, *GRB2* growth factor receptor-bound protein 2, *HES* hairy enhancer-of-split, *HEY* hairy and enhancer of split related protein, *HIF-1alpha* hypoxia inducible factor 1 alpha subunit, *LANA* latency-associated nuclear antigen, *MAML* mastermind-like protein, *MEK* MAPK kinase, *mTOR* mammalian target of rapamycin, *MYC* Myc proto-oncogene, *PLCG1* phospholipase C-gamma 1, *PI3K* phosphoinositide 3-kinase, *PTPRB* receptor-like protein-tyrosine phosphatase (PTP) beta, *RBPJ* recombinant binding protein suppressor of hairless, *S6K* P70-S6Kinase 1, *TACE* ADAM17, *Tie2* TEK tyrosine kinase, endothelial, *Tsc1* tuberous sclerosis 1, *VEGF(R)* vascular endothelial growth factor (receptor)

A common feature of endothelial cell malignancies is the dysregulation of normal endothelial cell signaling pathways (Fig. 2). Multiple mechanisms contribute to the observed dysregulation, including viral oncoproteins, chromosomal rearrangements, and paracrine signaling in the microenvironment. To date, targeting angiogenesis pathways has had varying degrees of success (Table 2). In this review, we summarize the existing clinical, molecular, and biological knowledge to frame a path toward a greater understanding of the pathophysiology of endothelial cell malignancies and improved clinical outcomes.

**Table 2:** Current status of drugs targeting deregulated signaling pathways in angiosarcoma and other endothelial cell neoplasms

Target	Drugs	Current development status
Angiogenesis		
VEGF	Bevacizumab	Phase 2 trials with RR 9% (ref. 22) and 6 month PFS 54%, median OS 19.5 months;^23^ no benefit over paclitaxel alone
	Aflibercept	No published studies to date
VEGFR2	Sorafenib	Phase II with RR 0–14%, median PFS of 2–5 months^24–26^
	Sunitinib	Case reports^29, 30, 35^ and 2 angiosarcoma patients in large Phase 2 trial^31^
	Pazopanib	Case reports,^27, 28^ Phase 2 trial ongoing (NCT01462630)
	Cediranib, Axitinib, Ramucirumab	No published studies to date
Tie2/Ang2	Trebananib	Phase 2, no responses in 16 patients^46^
Notch/DLL4	Gamma-secretase inhibitors	GEM Notch KO models develop hepatic angiosarcomas,^52, 53^ no clinical studies in angiosarcoma; Preclinical studies with gamma-secretase inhibitors with activity against KS cell lines^126^
FGF	Anti bFGF oligonucleotides	In vitro activity in KS^132^
	Sunitinib	See sunitinib above
PDGF/PDGFR	Imatinib	Case reports^59^
	Dasatinib	In vitro activity in canine angiosarcoma^57^
	Olaratumab	Phase II trial in soft tissue sarcoma, including angiosarcoma (NCT01185964)
Angiostatin/endostatin	Angiostatin/endostatin	Case report,^62^ preclinical activity in hemangioendothelioma^64^
CD105 (endoglin)	TRC0105	Phase 1b/2a combined with pazopanib (NCT01975519)
Intracellular kinase and mTOR pathways		
PLCG1	No published inhibitors in clinical development	
Raf	Vemurafenib, dabrafenib	No published studies to date
MEK	Trametinib, cobimetinib	Preclinical activity in canine angiosarcoma with single agent and combined MEK and mTOR inhibition^88^
mTOR	Everolimus, sirolimus, temsirolimus	Preclinical studies in angiosarcoma (see above); Case report in EHE;^174^ Tumor regression in transplant associated KS,^128^ phase I study in KS combined with HAART^129^
Transcriptional control		
MYC	BET inhibitors	Not currently being tested in vascular tumors
Beta-receptors	Propranolol	First-line therapy for IH;^175–177^ preclinical studies showed synergy in angiosarcoma and EHE cell lines^178^
Glucocorticoid receptors	Prednisolone, methylprednisolone	First-line for hemangioendothelioma associated with Kasabach-Merritt syndrome^179^
	Prednisolone	RR 90% in IH^146, 147^
	Prednisone	Similar response rate to propranolol in a phase 2 study for proliferating IH^180^

*VEGF* vascular endothelial growth factor, *RR* response rate, *PFS* progression free survival, *OS* overall survival, *VEGFR2* vascular endothelial growth factor receptor 2, *Tie2* TEK tyrosine kinase, endothelial, *Ang2* angiopoietin 2, *DLL4* delta-like 4, *KS* Kaposi sarcoma, *FGF* fibroblast growth factor, *PDGF/PDGFR* platelet-derived growth factor/ platelet-derived growth factor receptor, *CD105* endoglin; mTOR mammalian target of rapamycin, *PLCG1* phospholipase C-gamma1, *Raf* Raf-1 proto-oncogene, *MEK* MAPK kinase, *EHE* epithelioid hemangioendothelioma, *HAART* highly active anti-retroviral therapy, *MYC* Myc proto-oncogene, *BET* bromodomain and extra-terminal domain family, *IH* infantile hemangioma

### Physiologic angiogenesis pathways in endothelial cell malignancies

#### Vascular endothelial growth factor (VEGF)/VEGF receptor (VEGFR) signaling

VEGF/VEGFR2-mediated signaling is critical for tip cell selection and migration in physiological angiogenesis. VEGFR2 and VEGFR3 both promote tip cell formation, breakdown of the basement membrane, and loss of pericyte coverage, allowing for endothelial cell migration. Unsurprisingly, endothelial cell neoplasms have aberrant VEGF/VEGFR pathway signaling.

In aggressive angiosarcomas, alterations in VEGF and its receptors have been well characterized, including mutations and amplifications (Fig. 2). Angiosarcomas have high VEGF-A and VEGFR (1–3) expression, with rates ranging from 65–94% for VEGFR (1–3).^10^ *VEGFR2* mutations have been reported in 10% of angiosarcomas^11^ and in 2 of 6 angiosarcomas in a smaller series,^12^ with mutations identified in the extracellular, transmembrane, and kinase domains. However, the prevalence of these *VEGFR2* mutations in angiosarcomas remains uncertain, as no *VEGFR2* mutations were revealed in other studies including whole-genome- or whole-exome sequencing.^13^ The functional consequence of these mutations is not fully understood, but at least some are thought to be activating mutations that act as drivers in a subset of angiosarcomas.^11^

Perhaps the strongest clinical correlation of VEGF/VEGFR dysregulation in angiosarcoma is the finding of *VEGFR3* (*FLT4*) gene amplification in secondary (radiation- or lymphedema- induced) angiosarcomas.^7^ These amplifications are generally found in combination with other alterations such as *c-MYC* amplification and mutations in *PLCG1* and *PTPRB*. Thus, although *VEGFR3* drives lymphangiogenesis and induces sprouting and tip cell migration, the individual contribution of *VEGFR3* amplification in these cases remains unclear. Effective targeting of the VEGF/VEGFR axis either by knocking down VEGF-A, -C, or -D, or treatment with the pan-VEGFR inhibitor axitinib in a mouse model with constitutive mammalian target of rapamycin (mTOR) activation (develop tumors consistent with lymphangiosarcoma) suggests that targeting the upstream ligand or receptor may be a therapeutic option even in cases when downstream activating mutations are identified.^14^ Although these alterations do not represent the primary genetic driver events, targeting VEGF/VEGFR signaling is a rational clinical approach in angiosarcoma.

VEGF signaling also contributes to the development of KS. AIDS-KS spindle cells are stimulated to secrete VEGF by platelet-derived growth factor (PDGF)-beta and interleukin (IL)-1 beta, and co-injection of these two factors increases the vascularity of KS-like lesions in mice.^15^ VEGF acts as an autocrine growth factor in KS, which has high levels of VEGFR1, VEGFR2,^16^ and VEGFR3^17^ compared with adjacent normal skin. Expression of the virally encoded vGPCR (discussed below) is sufficient to induce VEGF-mediated angiogenic phenotypic switching in mouse fibroblasts^18^ and immortalizes human umbilical vein endothelial cells (HUVEC) by upregulation and activation of VEGFR2^19^ VEGF expression is less well-characterized in other malignancies of endothelial cell origin. Some studies have reported VEGF expression in a high proportion of EHEs, and associated increased VEGF staining intensity with more aggressive disease.^20, 21^ Additional work is needed to fully characterize the contributions of the VEGF pathway in EHE pathogenesis.

Clinical results with anti-VEGF drugs such as bevacizumab and VEGFR2-blocking tyrosine kinase inhibitors (TKIs) have been disappointing in patients with endothelial cell malignancies (Table 2). The response rate to bevacizumab monotherapy was 9% (median progression free survival (PFS) of just 3 months) in patients with angiosarcoma;^22^ addition of bevacizumab to paclitaxel did not provide additional benefit over paclitaxel alone.^23^ Sorafenib, a TKI with anti-VEGFR2 activity, had a similarly low response rate reaching 14.6% in patients with advanced angiosarcoma (median PFS of 2–5 months).^24–26^ Clinical responses to other TKIs including pazopanib^27, 28^ and sunitinib^29, 30^ have also been published in small reports, but larger phase II and III clinical trials of sunitinib^31^ or pazopanib^32^ in soft tissue sarcoma included only a limited number of patients with angiosarcomas. Similarly, some studies have reported benefit in EHE patients treated with bevacizumab, sunitinib, or pazopanib, but larger studies are needed to draw definitive conclusions about response rates. In addition, these studies lacked a control arm, making it difficult to quantify the benefit of these agents in this indolent tumor with slow progression.^22, 33–35^ Interestingly, in patients with KS, HAART plus bevacizumab resulted in an overall response rate of 31%, with three complete responses.^36^ Despite this finding, anti-VEGF/VEGFR therapy is not currently used routinely in KS likely due to higher response rates with liposomal doxorubicin and taxanes.

#### Angiopoietin/Tie2 signaling

In contrast to VEGF signaling, which stimulates early vascular development, angiopoietin (Ang)/Tie signaling promotes endothelial cell survival and stability.^37^ Different Tie2 ligands, notably Ang1 and Ang2, have variable effects on Tie2 signaling. Ang1 is produced by multiple cell types, whereas Ang2 is produced primarily by endothelial cells and is expressed only in tissues undergoing remodeling.^38^ Ang2 can function as an agonist or antagonist depending on the environment.^39^ Interestingly, Ang2 is upregulated in solid tumor angiogenesis, and high levels of Ang2 are associated with worse outcomes in multiple cancer types.^40^

A transmembrane phosphatase, receptor-like protein-tyrosine phosphatase (PTP) beta (PTPRB), dephosphorylates Tie2, rendering it inactive.^41, 42^ Loss-of-function mutations in *PTPRB* are relatively common in angiosarcoma and were found in 26% of angiosarcomas; interestingly, all these mutations were in secondary angiosarcomas.^13^ Normally, PTPRB dephosphorylates Tie2; thus, loss of PTPRB in angiosarcoma likely increases Ang/Tie2 signaling and may activate multiple pathways downstream from Tie2, such as the protein kinase B (AKT)/phosphoinositide 3-kinase (PI3K)/mTOR, endothelial nitric oxide synthase, focal adhesion kinase (FAK), mitogen-activated protein kinase (MAPK), and downstream of kinase-related protein/non-catalytic region of tyrosine kinase adaptor protein 1/p21-activated protein kinase (DOCK/NCK/PAK) pathways.^40^ Silencing PTPRB in HUVECs led to increased sprouting, even in the presence of VEGFR2 inhibitors such as sunitinib, demonstrating that PTPRB loss represents a mechanism of canonical activation of Tie2 signaling in angiosarcoma.^13^

Ang2 is highly expressed in angiosarcoma, and higher Ang2 secretion is correlated with more advanced angiosarcoma stage; in contrast, Ang1 seems to have a minimal role in angiosarcoma^43^ and higher Ang1 expression correlates with improved survival.^44^ Ang2, Tie1, and Tie2 are strongly expressed in both angiosarcoma and KS samples.^44, 45^ The correlation of increased Ang2 expression with worse outcomes and that of increased Ang1 expression with improved survival suggests that these ligands may be promising therapeutic targets. However, in a phase II study of trebananib, a peptibody against both Ang1 and Ang2, no responses were seen in angiosarcoma patients. Trebananib increased Ang1 and Ang2 levels after treatment, likely contributing to the lack of response.^46^ Additionally, antagonizing Ang1 may diminish its seemingly beneficial effect. Given the differential effects of Ang1 and Ang2, a better approach may be to target Ang2 independently with a specific agent.

#### Notch signaling

In physiological vascular development, Notch signaling is active at endothelial tip cells, where increased DLL4 expression stimulates Notch signaling in neighboring stalk cells and inhibits them from migrating, thus promoting organized vascular branching.^47^ Upon ligand binding, Notch receptors are cleaved by regulated proteolysis and the cleaved intracellular component travels to the nucleus and interacts with hairy and enhancer of split related-2 (HERP-2/Hey-1) and hairy enhancer-of-split (HES) proteins, leading to transcription (Fig. 2).^48^ The Notch ligand Jagged1 has a pro-angiogenic effect, whereas DLL4 counters the proliferative effects of VEGF^49^ and Jagged1.^50^

The observation of endothelial neoplasms forming after DLL4 inhibition in mouse models implicated Notch signaling in the development of proliferative vascular tumors, especially in the liver.^51^ Conditional knockout of Notch1 in the liver in a mouse model resulted in hepatic angiosarcomas in 86% of mice by 50 weeks.^52^ Studies in a separate mouse model confirmed that loss of Notch1 heterozygosity leads to endothelial cell neoplasms of varying histological grade with approximately the same penetrance; interestingly, the liver was the primary site.^53^ However, few, if any, significant Notch abnormalities have been identified in the sequencing efforts of human angiosarcoma samples, and the clinical relevance of these observations remains to be determined. Notch pathway targeted therapies such as DLL4 inhibitors and Notch receptor antagonists have effects that would mechanistically lead to disorganized vascularization as seen in the mouse models, and are, therefore, not being tested in angiosarcoma. In other cancer types, Notch has oncogenic or tumor suppressor roles depending on context, and it similarly can have pro- or anti--angiogenic effects in physiological tissue remodeling. The potential effect of nonspecific Notch inhibition with gamma-secretase inhibitors on hemangioendothelioma and angiosarcoma remains to be determined. A pathogenic role for the Notch pathway, with druggable targets for therapeutic development, is more established in KS tumorigenesis primarily due to the effects of KSHV proteins on endothelial cells. This is described in more detail below. While these observations reflect the importance of this pathway in deregulation of angiogenesis, targeting Notch signaling is complicated by several unique features of this pathway. Effects of Notch are remarkably context dependent and the signal itself has dose-dependent effects downstream. Furthermore, Notch signal tends to have a very short intracellular half-life and sustained inhibition may not be needed. To utilize Notch inhibitors in treatment of angiosarcoma, it would be important to identify the optimal level and timing of inhibition for disease control without excessive toxicity.

### Other angiogenesis-related pathways in endothelial cell malignancies

#### Platelet-derived growth factor

Paracrine signaling between endothelial cells and perivascular cells is mediated, in part, by PDGF signaling. Endothelial cells secrete PDGF-BB, which increases pericyte coverage and maintains the integrity of the endothelial cell basement membrane.^54^ At least five different PDGF isoforms interact with two different PDGF receptors (PDGFRs).^55^ PDGFR activation results in autophosphorylation of the receptor, which in turn activates phospholipase C-gamma (PLCG).^56^ As described below, PLCG1 activation by VEGFR2 acts as a driver for a subset of angiosarcomas and leads to resistance to VEGF/VEGFR targeted therapies. Treatment with dasatinib or imatinib, which inhibit PDGFR as well as other kinases, decreased cell viability in vitro and decreased tumor growth in vivo in a canine xenograft model of hemangiosarcoma.^57^ Isolated responses to imatinib, a PDGFR inhibitor, have been noted in patients with angiosarcoma.^58, 59^ A recently completed clinical trial of olaratumab, a monoclonal antibody against PDGFR-alpha, combined with doxorubicin (NCT01185964) for soft tissue sarcoma showed promising results including an improvement in overall survival; olaratumab has not been evaluated for treatment of angiosarcoma. Endothelial cell malignancies contain disorganized endothelium, and have not been proven to be associated with pericytes in the same way as normal endothelial cells.

#### Angiostatin and endostatin

Angiostatin and endostatin are protein fragments that suppress tumor growth and angiogenesis.^60, 61^ Endostatin has been used to treat angiosarcoma, but its effectiveness could not be determined because it was given in combination with cytotoxic chemotherapy.^62^ The vast majority (>90%) of endothelial cell neoplasms including benign hemangiomas, EHEs, and angiosarcomas express annexin II, an angiostatin receptor.^63^ Angiostatin inhibits hemangioendothelioma growth in vivo, but does not affect proliferation or induce apoptosis in vitro.^64^ Interestingly, endostatin paradoxically stimulates hemangioma-derived endothelial progenitor cells in an in vitro migration assay, a phenomenon not observed in hemangioendothelioma cells.^65, 66^ Although angiostatin and endostatin are sometimes used in patients with endothelial cell malignancies, more evidence is needed to fully assess their potential benefits.

#### Endoglin/transforming growth factor beta

Endoglin (CD105) is a component of the transforming growth factor beta receptor family that is expressed on endothelial cells, mesenchymal stem cells, and monocytes and has been specifically considered a drug target for novel agents designed to target tumor angiogenesis.^67, 68^ In physiological angiogenesis, endoglin mediates TGF-beta signaling via activin a receptor type II-like 1 (ALK1), which acts as a pro-angiogenic mediator and increases endothelial cell migration and proliferation, counteracting the potential inhibitory effect of TGF-beta on endothelial cells.^69^ There are two isoforms of endoglin, with S-endoglin playing a critical role in vascular senescence.^70^ Endoglin mutations cause hereditary hemorrhagic telangiectasia type 1, which is characterized by vascular dysplasia and hemorrhage,^71^ but to date no mutations in endoglin have been identified in endothelial cell neoplasms. Angiosarcomas have high expression levels of endoglin, with 95–100% staining positive.^72, 73^ Levels of TGF-beta pathway proteins are higher in angiosarcoma of bone than in primary angiosarcomas of soft tissue.^74^ However, the importance of endoglin in mediating the observed increase in TGF-beta signaling in bone angiosarcoma is not established, and the near universal expression of endoglin in angiosarcomas regardless of their site of origin makes any causative presumptions premature without additional study. An anti-endoglin antibody is currently being tested in clinical trials in combination with pazopanib for soft tissue sarcoma, with preliminary results in a phase I/II trial showing 2/2 patients with complete response, suggesting efficacy in angiosarcoma that requires further investigation (NCT01975519).^75^

### Intracellular oncogenic signaling pathways

#### Mitogen-activated protein kinases

In normal endothelial cells, VEGF induced VEGFR endocytosis and regulated MAPK activation are important factors that stabilize filopodia-carrying endothelial sprouts and ensure that a transient signal allows for stability in a branching vessel.^76^ VEGFR2 activation leads to PLCG1 phosphorylation and transduces the activating signal of its binding ligand to ensure normal vascular function.^77–80^ Autophosphorylation of VEGFR2 leads to the recruitment of PLCG1, binding of PLCG1 at its N-terminal SH2 domain, and subsequent activation of PLCG1 (ref. 81). Phosphorylation at PLCG1-Y783 causes a conformational change that relieves the auto-inhibition of the C-terminal SH2 domain, and leads to downstream signaling.^82, 83^ Activated PLCG1 catalyzes the conversion of phosphatidylinositol 4,5-bisphosphate to diacylglycerol and inositol 1,4,5-triphosphate, leading to protein kinase C-dependent MAPK signaling.^84, 85^ A recurring mutation in PLCG1, R707Q, has been identified in angiosarcoma, with a prevalence of 9–30% (refs 12, 13). R707 is in the auto-inhibitory SH2 domain of PLCG1, and the missense mutation is hypothesized to destabilize this domain and reduce its auto-inhibitory effect.^13^ Indeed, canonical activation was demonstrated by expressing the PLCG1-R707Q in HUVECs, which resulted in the downstream activation of MAPK and NFAT-mediated signaling irrespective of PLCG1 activation by VEGFR2 (ref. 12). Interestingly, sequencing from a patient with progression following initial response to sunitinib revealed a PLCG1-R707Q mutation in the metastasis, but this mutation was not found in the primary tumor. This finding suggests a mechanism by which angiosarcomas could develop adaptive resistance to VEGFR-targeted therapy.^86^ In addition to PLCG1 mutations, mutations or amplifications in *K-, H-, and N-RAS, B- and C-RAF, and MAPK1* were identified in angiosarcoma.^87^ Preclinical studies with canine angiosarcomas have demonstrated anti-tumor activity with MEK inhibition.^88^

#### Phosphoinositide 3-kinase/protein kinase B/mammalian target of rapamycin

The PI3K/AKT/mTOR signaling pathway is frequently activated in many cancer types. In normal endothelial cells, this pathway is activated through several stimuli, including VEGF, Ang/Tie2, and integrins.^89^ AKT is phosphorylated compared to normal adjacent endothelial cells in nearly all endothelial cell neoplasms.^90, 91^ Interestingly, different AKT isoforms have opposing effects. In hemangioma, hemangioendothelioma, and angiosarcoma models, AKT1 has been demonstrated to promote growth and migration, whereas AKT3 inhibits growth.^90^ Overexpression of AKT1 is sufficient to create proliferative neoplasms, but without the ability to metastasize.^92^

Downstream in the PI3K pathway, mTOR complex 1 (mTORC1) activation leads to the activation of p70 S6-kinase and S6 ribosomal protein. Angiosarcomas have increased activation of S6-kinase and S6 ribosomal protein; topical rapamycin inhibits the growth of patient-derived infantile hemangioma (IH) cells and established hemangioendothelioma and angiosarcoma cell lines in vitro and the growth of hemangioendothelioma mouse xenografts.^93^

Mice with a conditional knockout of tuberous sclerosis complex 1 (Tsc1), a negative regulator of mTORC1, develop paw angiosarcomas at 6 weeks that are responsive to rapamycin.^94^ The sustained mTORC1 signaling that results from Tsc1 loss leads to increases in HIF1-alpha and c-MYC-mediated VEGF transcription, creating an autocrine loop that is required for tumor maintenance.^14^ Although canine angiosarcoma xenografts in athymic nude mice showed minimal response to temsirolimus alone, temsirolimus sensitized angiosarcomas to MEK inhibition, suggesting cross-talk between the MAPK and PI3K/AKT/mTOR pathways.^88^ Although the PI3K/AKT/mTOR pathway is activated in angiosarcoma, this activation does not appear to be due to mutations or losses directly affecting this pathway, such as phosphatase and tensin homolog loss or PI3K mutations.^95^ Targeting the pathway with mTOR inhibitors remains a promising therapy and is currently used for patients with angiosarcoma in the investigational setting.

### Transcriptional regulation

#### Myc proto-oncogene (MYC)

Dysregulation of the transcription factor MYC has been implicated in many cancer types.^96^ *MYC* amplification is well described in secondary angiosarcoma^7, 97^ and has also been demonstrated in primary angiosarcoma.^98^ In addition to MYC, Ret proto-oncogene (*RET*) signaling is upregulated and cyclin-dependent kinase inhibitor 2C is downregulated in secondary angiosarcoma,^99^ which is in agreement with the finding that N-MYC is a downstream target of Ret that downregulates cyclin-dependent kinase inhibitor p18, leading to proliferation of cultured fibroblasts.^100^ MYC may contribute to the aggressive angiogenic phenotype of angiosarcoma by upregulating miR-17-92 and thus downregulating thrombospondin-1 (TSP1), an angiogenesis inhibitor.^101^ Genomic analyses revealed that MYC-related pathways were upregulated in murine angiosarcoma cell lines isolated from primary hepatic angiosarcomas in *Notch1* conditional knockout mice.^102^ Interestingly, mice in which Cdk6 activity is increased by rendering them insensitive to inhibition by INK4 develop angiosarcomas with a high prevalence (~50%), particularly when the Cdk6 alteration is introduced in a p53 heterozygous or null background.^103^ To date, Cdk4/6 inhibitors have not been evaluated in angiosarcoma, but could represent an important opportunity.

MYC also plays an important role in KS pathogenicity. The KSHV virus protein LANA stabilizes c-MYC by preventing its phosphorylation at Thr58, thus preventing apoptosis.^104^ In primary effusion lymphoma, another malignancy associated with KSHV, MYC is required to maintain the latency of KSHV,^105^ and inhibition of bromodomain and extra-terminal domain bromodomain (BET), a therapeutic strategy for targeting pathologic MYC activation,^106^ has had promising results in vitro in other KSHV associated tumors.^107^ In other models, MYC has been shown to promote neovascularization by either downregulating anti-angiogenic factors such as TSP1 and connective tissue growth factor^108^ or interacting with hypoxia to induce VEGF-A production.^109^ The latter mechanism, which used a model for dermal angiogenesis, may be particularly relevant in endothelial cell tumors that commonly arise in the skin.

#### HIF/hypoxia

Under normoxic conditions, the transcription factor HIF-1a is primed for degradation by the von Hippel-Lindau tumor suppressor (VHL), an E3 ubiquitin ligase. Hypoxia leads to HIF-1a-mediated pro-angiogenic signaling and consequently the recruitment of blood vessels to solid tumors. In mice, removal of VHL’s inhibition of HIF via mosaic VHL knockout led to the formation of vascular lesions ranging from hemangiomas to a single mouse that developed an angiosarcoma.^110^ HIF-1a and HIF-2a expression is reported in a subset of angiosarcomas,^111, 112^ but HIF-1a does not appear to be a notable driver of angiosarcoma growth.^112^

On the other hand, HIF-1a and hypoxia-related pro-angiogenic pathways play a role in the transition of KSHV-infected endothelial cells to KS.^113^ A G protein-coupled receptor encoded by KSHV (vGPCR) contains an activating V138D mutation, which leads to agonist-independent induction of the MAPK and p38 signaling pathways. This, in turn, leads to HIF-1a phosphorylation and a HIF-1a-dependent increase in VEGF secretion.^114^ In addition, vGPCR also increases mTOR complex signaling, suggesting that multiple pathways activated by vGPCR converge on HIF-1a-mediated VEGF transcription and secretion.^115^ HIF-1a-mediated transcription is also induced by the KSHV LANA protein, by targeting the HIF-1a suppressors VHL and p53 for degradation,^116^ as well as by direct protein–protein interactions between LANA and HIF-1a that stabilize HIF-1a and promote its translocation to the nucleus (Fig. 2 ^117^).

### Viral oncoproteins in KS

The discovery of KSHV led to discoveries regarding the oncogenic role for the virus (reviewed in ref. 118). Here, we focus specifically on KSHV and its direct role in co-opting physiologic angiogenesis to lead to transformation of endothelial cells to Kaposi spindle cells.

#### Kaposi sarcoma-associated herpes virus

Like other herpes viruses, KSHV infection consists of two phases: the lytic phase in which the virus infects the host cell and replicates and the latent phase in which the viral DNA remains in the host cell but is not actively replicating. Viral proteins specific for both phases directly interact with components of angiogenic signaling to promote tumorigenesis. Specifically, the latency associated proteins LANA and vFLIP induce Notch ligands Jagged1, and DLL4. LANA stabilizes Hey1,^119^ leading to decreased Hey1 degradation and consequently increased endothelial cell proliferation.^120^ Furthermore, KSHV harnesses Notch signaling to induce its lytic phase by utilizing RBP-J, a key transcription factor for Notch related genes, to initiate transcription of its lytic phase genes.^121^ vIL-6, a lytic phase protein, induced expression of Notch4, DLL1, DLL4, and downstream targets Hey1 and Hey2, and vGPCR expression induced Notch2, Notch3, and Jagged1.^122^ Importantly, Notch3 is typically seen on mural or smooth muscle cells adjacent to endothelial cells but not in endothelial cells themselves.^123^ Induction of Notch3 suggests that KSHV induces a change in phenotype from differentiated endothelial cells. This is further supported by the observation that activation of the Notch-induced transcription factors Slug and zinc finger E-box-binding homeobox 1 after KSHV infection contributes to the endothelial-to-mesenchymal transition (EndMT) that is important for the malignant progression of infected endothelial cells independently from TGF-beta signaling,^124^ which regulates EndMT in non-malignant ECs.^125^ In vitro Notch inhibition with gamma-secretase inhibitors in KS-like cell lines induces mitotic catastrophe,^126^ suggesting that targeting Notch may be effective for KS. KSHV GPCR also stimulates MAPK signaling. Inhibition of the MAPK pathway in in vitro models of KS led to substantial reductions in the KSHV mediated induction of Notch pathway components, suggesting that MAPK signaling is the primary mechanism by which vGPCR induces Notch activation.^122^

In spite of the evidence showing the role of Notch and MAPK signaling in KS, the most clinical success to date has been by targeting the mTOR signaling. vGPCR directly leads to the overactivation of the PI3K/AKT/mTOR pathway that is necessary for KSHV-induced transformation of endothelial cells to KS spindle cells.^115, 127^ In addition, vGPCR activation of MTORC1 leads to secretion of pro-angiogenic factors causing paracrine signaling, which recruits and stimulates other non KSHV infected cells.^127^ As a result, mTOR inhibition has been used more successfully in the clinic for KS. Among kidney transplant patients with KS as a result of prolonged immunosuppression with cyclosporine, changing the drug to sirolimus resulted in rapid regression of all cutaneous KS lesions.^128^ Furthermore, long-term stabilization with mTOR inhibition was also seen in AIDS-related KS,^129^ demonstrating the importance of pathogenic KSHV viral signaling even in co-infected patients.

#### Human immunodeficiency virus

Overexpression of fibroblast growth factor (bFGF) in normal endothelial cells results in vascular tumors in mice.^130^ In endothelial cell cancers, FGF signaling plays the largest role in KS. Synergy between the HIV-1 Tat protein and bFGF promotes the development of an angiogenic malignant phenotype in a preclinical mouse model.^131^ Targeting bFGF with antisense oligonucleotides inhibits the growth of AIDS-KS cells in vitro and in vivo, suggesting that targeting bFGF is a potential therapeutic strategy for KS.^132^ Tat works by several mechanisms, including binding to alpha-5-beta-1 and alpha-v-beta-3 integrin receptors via its RGD domain and stimulating migration and invasion, and also stimulating the release of preformed, extracellularly bound bFGF into a soluble form that can induce vascular cell growth and prevent apoptosis.^133–135^ Tat can also directly bind to VEGFR2 and stimulate VEGFR2 signaling independent of VEGF-A.^136^ Downstream, Tat activates multiple growth promoting signaling pathways including MAPK and FAK.^137^

### Microenvironment and intercellular interactions

The complex interplay between various components of the tumor microenvironment (e.g., immune cells, fibroblasts, endothelial cells) can have either pro- or anti-tumor effects depending on the specific circumstances. Among patients with angiosarcoma, the presence of CD8 + tumor infiltrating lymphocytes correlates with a survival advantage.^138^ In addition to CD8 + cells, endothelial malignancies also have infiltration of CD3 + and CD4 + lymphocytes, as well as regulatory FoxP3 lymphocytes. In EHE, high CD3 + and FoxP3 + lymphocytes but not CD4 + or CD8 + lymphocytes were noted. All of the vascular tumors had high macrophage infiltration.^139^ Conflicting data exist regarding the expression of programmed death-ligand 1 (PD-L1).^139, 140^

Macrophages, in particular tumor-associated macrophages, have long been known to have a pro-angiogenic effect that promotes tumor survival (reviewed in ref. 141). Macrophages are recruited to tumors by chemoattractants produced by tumor cells. A series of studies from Japan investigated the use of risedronate (to target macrophages) combined with cytotoxic chemotherapy in the adjuvant setting with promising results.^142, 143^ High levels of Foxp3 + regulatory T-cells and CD163 + inhibitory macrophages relative to the number of cytotoxic T-cells were seen in these patients with primary angiosarcoma.^143^ In vitro treatment of angiosarcoma derived macrophages with docetaxel and risedronate increases the expression of C-X-C motif chemokines 10 and 11 (CXCL10 and CXCL11), both chemokines that recruit cytotoxic T-cells, in the treated macrophages.^144^ In a mouse model of angiosarcoma, inhibition of tumor-secreted IL-6 decreased macrophage numbers and increased cytotoxic T-cell infiltration, thereby decreasing tumor growth.^145^ This IL-6 secretion is dependent on an autocrine/paracrine network by which inhibitor of nuclear factor kappa-B (NF-KB) kinase subunit beta (IKK-beta) leads to IL-6 production and increased Stat3 activation by NF-KB-mediated transcription of gp130 and Janus kinase 2. In addition to the direct effect of IKK-beta on angiosarcoma cells, knockout of IKK-beta in host myeloid cells decreased neutrophil-derived nitric oxide, increased IL-4, and decreased IL-12 and interferon (IFN)-gamma, thus shifting the myeloid cells to the N2/M2 phenotype and increasing angiosarcoma growth.^145^ Moreover, inhibition of tumor secreted IL-8 has little effect on angiosarcoma cells in vitro, but prevents engraftment in vivo.^146^

In addition to the immunosuppression required for HHV8 infection, the immune microenvironment itself contributes to the malignant transformation of endothelial cells into KS. For example, normal endothelial cells cultured in media conditioned with activated T-cells have a phenotype consistent with early KS and are tumorigenic in nudemice.^147^ Both AIDS-associated and classical KS are infiltrated by CD8 + T-cells and CD14 + /CD68 + monocytes and macrophages that produce IFN-gamma.^148^ IFN-gamma induces KS spindle cells with an angiogenic phenotype that are similar to early KS cells. In contrast to findings in other tumors suggesting that high levels of infiltrating CD8 + lymphocytes are associated with improved outcomes, these lymphocytes have been proposed to contribute to the development of KS by producing IFN-gamma locally in the microenvironment.

**Fig. 3:**
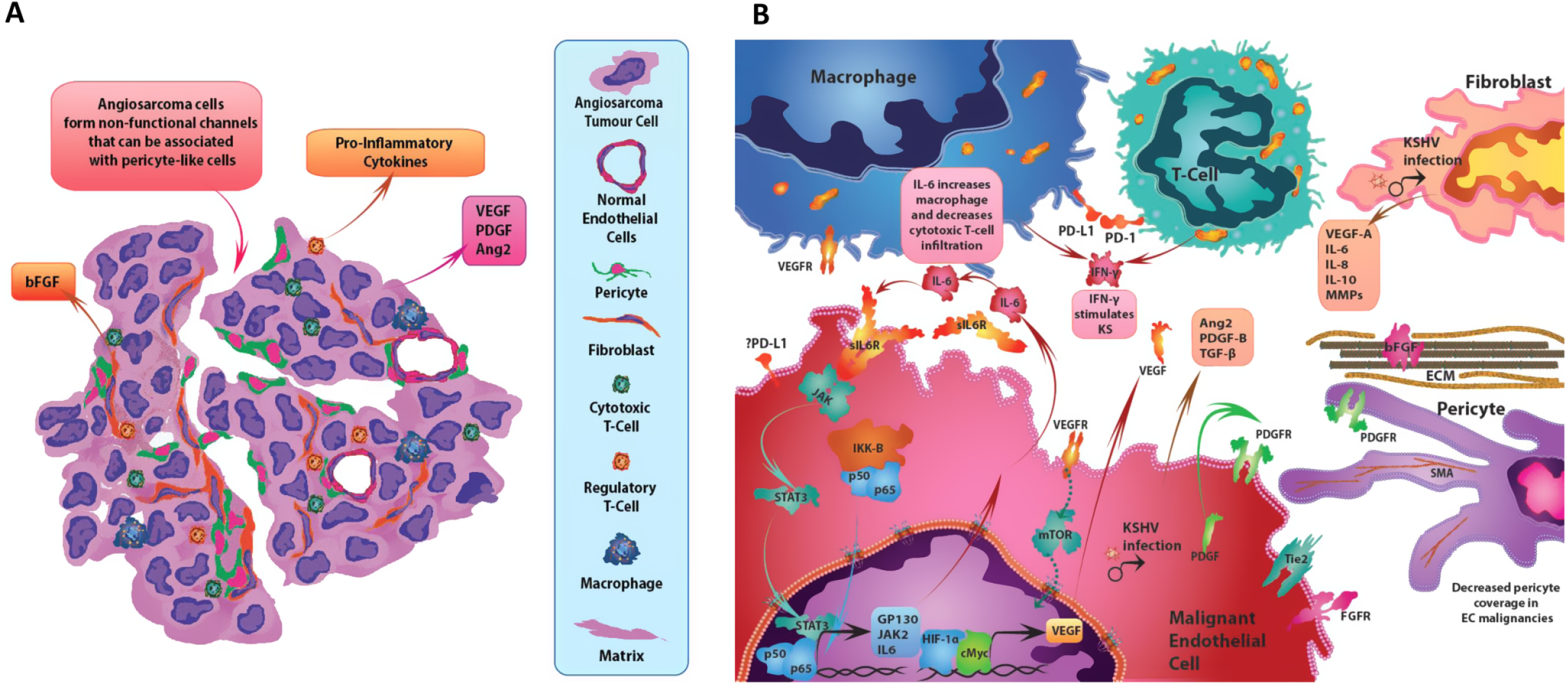
Illustration of microenvironment in endothelial malignancies. **a** Tissue level schematic of angiosarcoma composed of malignant endothelial cells that form non-functional channel like structures. Stromal support cells such as fibroblasts and pericytes, and immune cells such as macrophages, cytotoxic and suppressor T-cells, and non-malignant endothelial cells all interact to promote tumorigenesis and angiogenesis in the microenvironment. **b** A selection of autocrine and paracrine signaling networks in the microenvironment of endothelial malignancies. Pro-inflammatory cytokine interleukin-6 (*IL*-6) is secreted by angiosarcoma cells and is dependent on JAK/STAT and IKK-beta signaling. Interferon-gamma (*IFN-*gamma) is secreted by macrophages and cytotoxic T-cells, which stimulates Kaposi sarcoma (*KS*) tumor growth. Kaposi sarcoma herpes virus (*KSHV*) infection in resident fibroblasts stimulates secretion of multiple pro-angiogenic factors. Pericyte coverage is decreased compared to normal endothelium in endothelial malignancies, though the role of pericytes in directly promoting tumor growth is currently debatable. *Ang2* angiopoietin2, *b-FGF* basic fibroblast growth factor, *ECM* extracellular matrix, *FGFR* fibroblast growth factor receptor,*HIF-1alpha* hypoxia inducible factor 1 alpha subunit, *JAK* tyrosine protein kinase, *mTOR* mammalian target of rapamycin, *MMPs* matrix metalloproteinases, *MYC* Myc proto-oncogene, *PD1* programmed cell death protein 1, *PDGF(R)* platelet-derived growth factor (receptor), *PDL1* programmed cell death 1 ligand, *sIL6R* soluble IL6 receptor, *SMA* smooth muscle actin, *STAT* signal transducer and activator of transcription, *Tie2* TEK tyrosine kinase, endothelial, *VEGF(R)* vascular endothelial growth factor (receptor)

Pericytes have long been known to communicate with endothelial cells to maintain EC stability. A minority of angiosarcomas develop pericyte coverage.^149^ Angiosarcomas that stain positive for alpha-smooth muscle actin (alpha-SMA), a pericyte marker, tend to have positive staining around malignant non-functional vascular channels.^149, 150^ In physiological vascular regulation, pericytes slow endothelial growth and their loss in other cancer types correlates with increased metastasis.^151^ In contrast, pericytes derived from less aggressive endothelial cell tumors contribute to the pro-angiogenic microenvironment by constitutive expression of VEGF-A and decreased Ang1 secretion^152^ (Fig. 3). Further studies are needed to clarify the role of the observed pericyte-like cells in high-grade endothelial malignancies.

Finally, cancer-associated fibroblasts (CAFs) are activated stromal cells that have been shown to play a key role in promoting tumorigenesis in multiple cancer types. Interestingly, one of the primary mechanisms by which CAFs exert this effect is through VEGF^153^ and paracrine mediated crosstalk with endothelial cells.^154^ There is scant data regarding the role of fibroblasts specifically in endothelial cell malignancies, and the potential role of these cells needs to be investigated in angiosarcoma and hemangioendothelioma. The role of fibroblasts contributing to the paracrine growth signaling in KS is more established; latent infection of fibroblasts with KSHV leads the fibroblasts to secrete pro-inflammatory cytokines such as VEGF-A, IL-6, IL-8, and IL-10, as well as matrix metalloproteinases that break down the extracellular matrix and increase tumor cell invasion,^155, 156^ and are required by KS spindle cells for in vitro growth and to maintain their tumorigenic potential in nude mice.^157^

### Chromosomal rearrangements

Several chromosomal translocations are associated with vascular tumors (Table 1). Two separate translocations, both involving the Hippo pathway, were recently associated with EHE. The first, t(1;3), results in the fusion of the tafazzin gene *TAZ* (also known as *WWTR1*) and the calmodulin-binding transcriptional activator 1 gene *CAMTA1*.^158–161^ The second translocation results in the fusion of the yes-associated protein 1 gene *YAP1* and the transcription factor binding to IGHM gene *TFE3*.^162^ YAP and TAZ are transcription factors involved in the Hippo pathway and, in normal cells, are involved in regulating cell size. The role of YAP and TAZ in cancer was reviewed recently.^163^ In endothelial cells, endoglin activation leads to YAP translocation to the nucleus and induction of extracellular matrix remodeling and secretion of pro-inflammatory chemokines.^164^ An additional translocation involves chromosomes 10p13 and 14q24; this specific translocation may involve placental growth factor (PlGF) and serve as a driver of EHE in some patients.^165^

The pseudomyogenic hemangioendothelioma subtype is also associated with a balanced chromosomal translocation, t(7;19)(q22;q13), which results in a fusion of the serpin peptidase inhibitor, clade E (nexin, plasminogen activator inhibitor type 1), member 1 gene *SERPINE1* and FBJ murine osteosarcoma viral oncogene homolog B gene *FOSB*. This fusion is believed to lead to expression of the FOSB transcription factor under the *SERPINE1* promoter.^166^ Although VEGFR1 is generally considered a decoy receptor that sequesters VEGF from VEGFR2, PlGF was previously shown to induce *FOSB* transcription via VEGFR1 independent from VEGFR2.^167^

Most studies that looked at angiosarcoma cytogenetics identified complex cytogenetics.^12, 13, 168^ Aberrations included gain or loss of entire chromosomes as well as partial chromosomes; interestingly, 2 of 8 cases in one series had duplication of the region on chromosome 4q that contains *KIT* and *VEGFR2*.^12^ A fusion of the Nucleoporin 160 kDa gene *NUP160* and Solute Carrier Family 43, Member 3 gene *SLC43A3*, both on chromosome 11, was recently found in both primary angiosarcoma specimens and an established angiosarcoma cell line.^169^

## Conclusions and future directions

Endothelial cell malignancies are characterized by dysregulation in multiple pathways that are highly regulated in endothelial cells for normal vascular development, as well as by some of the more typical oncogenic pathways found in other cancers. Aberrant activation of other regulatory pathways may explain why the majority of these tumors do not respond to VEGF-targeted therapies. Patient-derived cell lines and model systems that better replicate the biology seen in human angiosarcomas and hemangioendotheliomas are urgently needed to further our understanding of these rare tumors. The currently available genetically engineered mouse models of angiosarcoma are driven by knockout of Notch pathway components or FoxO,^170^ or overactivation of the mTOR pathway, but these may not reflect findings in human angiosarcomas. Canine models exist, but these are not practical for large scale in vivo research.

Future investigation should focus on mechanisms of adaptive resistance in those with initial responses to angiogenesis inhibitors. Understanding the mechanisms by which vascular tumors have primary or adaptive resistance to anti-angiogenic therapies can guide future research and treatment paradigms not only for malignancies of endothelial cell origin, but also for other cancers (e.g., ovarian,^171^ lung,^172^ and colon^173^). Future studies should also focus on rational drug combinations to block oncogenic pathways, as well as evaluating combinations of targeted therapy with conventional modalities such as chemotherapy and radiotherapy. The low incidence of these tumors limits the amount of tissue available for clinical and correlative research. Modified clinical trial designs and increased multi-institutional collaboration are needed to ensure sufficient sample sizes and to accelerate clinical studies of these rare tumors.
